# Vitamin D: Evidence-Based Health Benefits and Recommendations for Population Guidelines

**DOI:** 10.3390/nu17020277

**Published:** 2025-01-14

**Authors:** William B. Grant, Sunil J. Wimalawansa, Pawel Pludowski, Richard Z. Cheng

**Affiliations:** 1 Sunlight, Nutrition, and Health Research Center, 1745 Pacific Ave., Ste. 504, San Francisco, CA 94109, USA; 2 Endocrinology & Human Nutrition, Department of Medicine, Cardiometabolic & Endocrine Institute, North Brunswick, NJ 08902, USA; suniljw@hotmail.com; 3 Department of Clinical Biochemistry, The Children’s Memorial Health Institute, 04-730 Warsaw, Poland; p.pludowski@ipczd.pl; 4 Orthomolecular Medicine News Service, Columbia, SC 29212, USA; 5 Low Carb Medicine Alliance, Shanghai 201613, China

**Keywords:** cancer, cardiovascular disease, chronic kidney disease, chronic lower respiratory diseases, COVID-19, dementia, diabetes mellitus, Endocrine Society, pregnancy

## Abstract

Vitamin D offers numerous under-recognized health benefits beyond its well-known role in musculoskeletal health. It is vital for extra-renal tissues, prenatal health, brain function, immunity, pregnancy, cancer prevention, and cardiovascular health. Existing guidelines issued by governmental and health organizations are bone-centric and largely overlook the abovementioned extra-skeletal benefits and optimal thresholds for vitamin D. In addition, they rely on randomized controlled trials (RCTs), which seldom show benefits due to high baseline 25-hydroxyvitamin D [25(OH)D] concentrations, moderate supplementation doses, and flawed study designs. This review emphasizes the findings from prospective cohort studies showing that higher 25(OH)D concentrations reduce the risks of major diseases and mortality, including pregnancy and birth outcomes. Serum concentrations > 30 ng/mL (75 nmol/L) significantly lower disease and mortality risks compared to <20 ng/mL. With 25% of the U.S. population and 60% of Central Europeans having levels <20 ng/mL, concentrations should be raised above 30 ng/mL. This is achievable through daily supplementation with 2000 IU/day (50 mcg/day) of vitamin D_3_, which prevent diseases and deaths. Furthermore, a daily dose between 4000 and 6000 IU of vitamin D_3_ to achieve serum 25(OH)D levels between 40 and 70 ng/mL would provide greater protection against many adverse health outcomes. Future guidelines and recommendations should integrate the findings from observational prospective cohort studies and well-designed RCTs to improve public health and personalized care.

## 1. Introduction

The new 2024 Endocrine Society guidelines have been published under the title “Vitamin D for the prevention of disease: An Endocrine Society clinical practice guideline” [1]. Despite the authors’ intention regarding whether this new document should replace the previous guidelines (2011) [2], it raises concerns about vitamin D and human health throughout life, from intra-uterine life until the oldest age. The 2024 Endocrine Society guidelines state that they are intended for clinicians but advocate against the measurement of 25-hydroxyvitamin D [25(OH)D], even in vulnerable groups, and against routine vitamin D empiric supplementation for disease prevention, except for children, pregnant women, pre-diabetic patients, and people aged 75 years and older. These new Endocrine Society guidelines were the impetus for the present review.

### 1.1. Global Vitamin D Deficiency

Vitamin D, often called the “sunshine vitamin”, is essential for many biological and physiological human processes. Despite its well-documented importance, vitamin D deficiency (VDD) remains a significant global public health issue. VDD is generally defined as serum 25(OH)D concentrations below 20 ng/mL [2]. An analysis of over 3.8 million measurements of 25(OH)D concentrations in the U.S. for 2007–2009 provided data on VDD in the U.S. [3]. The peak percentages of measurements of 25(OH)D_3_ < 20 ng/mL were 23% in winter and 44% in summer. However, many participants, especially in the northern states, took vitamin D_2_ supplements. As a result, the peak percentages of measurements of 25(OH)D concentration < 20 ng/mL were 15% in winter and 33% in summer. An analysis of deaths by day of the year from 1979 to 2004 in the U.S. found that rates were 30% higher near the end of the year than near the end of summer [4]. Furthermore, evidence has been reviewed supporting the hypothesis that a significant fraction of the increased number of deaths in winter could have been reduced by maintaining higher 25(OH)D concentrations [5].

Globally, the prevalence of VDD has been estimated as 45% (95% CI, 45–51%) for the period 2000–2022 [6]. As a function of latitude, the prevalence varied from 57% (95% CI, 45–70%) for latitudes 60–80° N to 18% (95% CI, 11–27%) for latitudes 20–60° N. As 25(OH)D concentrations below 20 ng/mL have the strongest correlations with adverse health outcomes, raising 25(OH)D concentrations above 20–30 ng/mL can be expected to have very significant health benefits.

This study reviews the myriad health benefits of vitamin D, supporting the need to update vitamin D-related clinical guidelines. It examines the limitations of current guidelines, specifically those issued by the Endocrine Society [1], which do not encompass the vitamin’s broader roles in health and disease prevention or treatment. Another set of guidelines with weak recommendations is that of the National Institute of Medicine’s Office of Dietary Supplements [7]. These guidelines recommend a supplementation of 600–800 IU/day of vitamin D and review the evidence from observational studies on vitamin D for various health outcomes, including cancers, cardiovascular disease (CVD), and type 2 diabetes mellitus (T2DM), finding that the evidence does not vary compellingly due to the lack of support from RCTs.

A consensus statement by 27 vitamin D researchers noted that post hoc analyses of RCTs revealed potential benefits in reducing the incidences of cancers, autoimmune diseases, cardiovascular events, and diabetes [8]. This statement acknowledges that VDD increases the risks of autoimmune and infectious diseases, cardiorespiratory diseases, impaired muscle function and strength, diabetes, cancer incidence and mortality, and acute COVID-19 severity, and long COVID risk. It also supports vitamin D supplementation with up to 2000 IU/day to achieve 25(OH)D concentrations between 30 and 50 ng/mL. Five vitamin D researchers—including two authors of the present review—recommended supplementation with 2000 IU/d vitamin D [9], which is considered sufficient to raise and maintain serum 25(OH)D concentrations above 20 ng/mL and 30 ng/mL in >99% and >90% of the general adult population, respectively.

### 1.2. Methodological Foundation

The research questions addressed in this review are as follows: what is the best evidence that vitamin D promotes good health through reducing the risk of the major vitamin D-sensitive diseases, and what serum 25(OH)D concentrations are associated with significantly reduced risks of these diseases?

Before reviewing the health benefits of vitamin D, it is helpful to explore how evidence for the beneficial effects of vitamin D was determined and which types of studies were included in this review. There are two main approaches for determining the health benefits of vitamin D: RCTs and observational studies. In RCTs, participants are assigned randomly to treatment or placebo groups, and health outcomes are compared with intention to treat. As discussed in the next section, vitamin D RCTs have largely failed due to their poor design, conduct, and analysis. However, a few RCTs have found beneficial effects of vitamin D supplementation, some of which are discussed in this review.

Observational studies use some measure of vitamin D, such as serum 25(OH)D concentration, oral vitamin D intake from food and/or supplements, or solar ultraviolet B (UVB) exposure, and include participants with large ranges of vitamin D exposure. As such, they often include large numbers of participants. While observational studies have some limitations (as discussed in Section 1.4), they often provide the best epidemiological support for vitamin D in reducing the risks and severity of disease and other health outcomes. Limitations include regression dilution due to long follow-up periods [10], concerns regarding generalization to other populations, and adjustments for confounding factors. Observational studies, primarily prospective cohort studies, form the basis for most of the recommendations provided in this review, and preference was given to meta-analyses of prospective cohort studies. Studies of the mechanisms of vitamin D are also instrumental in supporting the role of vitamin D in maintaining health and reducing the risks of disease incidence, severity, and mortality. Mechanisms are discussed for some of the health outcomes considered in this review.

The databases used in the searches for this review were Google Scholar and Pubmed.gov. Google Scholar was preferred, as it includes more journals than Pubmed.gov, shows which and how many other papers are cited in each paper, and it shows where to find an open-access version (if available). The search strategy was to search for papers with search terms including “vitamin D”, “supplementation”, “25-hydroxyvitamin D”, “risk”, “incidence”, “mortality”, “recommendations”, “RCTs”, “randomized controlled trials”, “observational study”, “cohort study”, “meta-analysis”, “recommendations”, “Mendelian randomization”, and “Hill’s criteria”. Preference was given to more recent and open-access publications.

For each disease, several representative papers are discussed. An effort was made to provide enough detail about each study or meta-analysis so readers could assess its relevance concerning the questions addressed.

### 1.3. Randomized Controlled Trials

Pharmaceutical companies use RCTs to obtain drug approval. In pharmaceutical drug RCTs, study participants are randomly assigned to treatment or control groups; only the treatment group receives the drug. Pre-defined clinical outcomes are compared through intention to treat analysis, compared to the placebo arm [11].

This approach is unsuitable and impractical for nutrients such as vitamin D, as there are many natural sources, and no individual is entirely vitamin D-depleted. Furthermore, vitamin D is a threshold nutrient, and the pharmaceutical study approach is unsuitable to test its efficacy [12,13]. In addition, most vitamin D RCTs have included participants with average serum 25(OH)D concentrations at or above 30 ng/mL, who may not benefit from vitamin D supplementation, depending on the body system under investigation. These RCTs have also generally provided the control arm with small doses of vitamin D and/or permitted them to take 600–800 IU/day of vitamin D—as recommended by the Institute of Medicine (IoM) [14]—based on ‘ethical’ concerns or mistakenly, as in two recent major vitamin D RCTs [15,16]. Unsurprisingly, RCTs have failed to support vitamin D’s role in reducing the risks of most diseases [17]. As discussed in recent reviews, this outcome is due to poor study designs, bias, conduct, and analysis of vitamin D in RCTs [12,18,19,20].

In 2014, Robert Heaney outlined guidelines for nutrient RCTs [21]. As applied to vitamin D, these guidelines strongly recommend measuring serum 25(OH)D concentrations in all prospective participants, and enrolling only those with low concentrations. Those in the treatment arm should be supplemented with sufficient vitamin D doses to raise their serum 25(OH)D concentrations to levels associated with significantly reduced risk [11,13]. The achieved mean serum 25(OH)D concentrations should be measured during the trial, and vitamin D doses should be adjusted as needed. Finally, the results should be analyzed regarding the 25(OH)D concentrations achieved. The only vitamin D supplementation study that comes closest to complying with Heaney’s guidelines is one evaluating the effects of vitamin D supplementation on pregnant women in Iran [22], which is discussed in detail later.

### 1.4. Observational Studies

As vitamin D is a threshold nutrient, RCTs are not the optimal way to test its efficacy [23]. A better way to ascertain the health benefits of vitamin D is through observational studies. Several types of observational studies, including geographical–ecological, prospective cohort, cross-sectional, and case–control studies, are most frequently performed. Geographical–ecological studies use data for populations in various geographical regions, generally using population-averaged data for health outcomes and risk-modifying factors. Such studies are often the first to identify vitamin D through solar UVB exposure as a risk reduction factor for diseases such as colon cancer [24]. The limitations of this approach include that the population-averaged data may not apply well to those with adverse health outcomes. Furthermore, important confounding risk-modifying factors may be overlooked.

Cross-sectional studies such as the National Health and Nutrition Examination Survey (NHANES) collect data from people representative of a population through interviews and physical examinations. An analysis of serum 25(OH)D concentrations measured in NHANES for the period 2003–2006 noted that measurements were made in the southern states in winter and northern states in summer due to the use of trailers [25]. Thus, some bias was introduced into the dataset. Case–control studies often compare variables between participants who have incident diseases with matched controls.

The best way to match cases and controls is by using propensity score matching. This approach was used in a case–control study of the effect of vitamin D on coronary atherosclerosis [26]. Cases and controls were matched for age, gender, smoking, arterial hypertension, positive family history, dyslipidemia, and diabetes. When propensity score matching is not used, cases and controls are likely not well matched. This concern has led to a distrust of case–control studies compared with prospective cohort studies.

Prospective cohort studies enroll large numbers of participants, collect data on many factors at the time of enrollment, and follow the participants for several years, noting changes in their health conditions. While widely used, they have a significant limitation in assessing the changes in essential factors such as serum 25(OH)D concentration over time, resulting in what has been termed “regression dilution” [10]. In a 1999 article, paired measurements of systolic blood pressure, diastolic blood pressure, and total cholesterol were recorded for participants in the Framingham Study (U.S.) over 30 years and the Whitehall Study (U.K.) over 26 years. The results showed that uncorrected associations between disease risk and baseline measurements underestimated the strength of the actual associations with usual levels of these risk factors during the first decade of exposure by about one-third, the second decade by about one-half, and the third decade by about two-thirds. This effect has been analyzed for prospective cohort studies regarding serum 25(OH)D concentrations, and it was found that, without accounting for the follow-up period, the beneficial effect against colorectal cancer was significantly underestimated for males using the traditional approach of averaging the results from all cohort studies, regardless of the mean follow-up period [27], as was further demonstrated in a review [28].

### 1.5. Pleiotropic Mechanisms

Most of the non-skeletal effects of vitamin D are produced through activation of the hormonal metabolite 1,25-dihydroxyvitamin D [1,25-(OH)_2_D or calcitriol]. The circulating metabolite, 25(OH)D, can be converted to calcitriol by the kidneys or other organs through the 25-hydroxylase CYP24A1 [29]. When calcitriol binds to vitamin D receptors coupled to chromosomes, it can affect the expression of many genes, upregulating some and downregulating others. A clinical study of healthy adults examined the number of genes up- or downregulated in white blood cells when supplemented with different vitamin D doses [30]. For doses of 600, 4000, or 10,000 IU/day for six months, the numbers of up- or downregulated genes were 162, 320, and 1289, respectively. This suggests that higher 25(OH)D concentrations lead to better health outcomes, which generally agrees with the findings of many studies.

The pleiotropic (genetic) mechanisms through which vitamin D reduces the risk of cancer incidence include those that reduce incidence—such as regulating cellular differentiation, proliferation, and apoptosis—and those that reduce progression through anti-angiogenesis mechanisms and mortality through reducing metastasis [28]. Vitamin D has various cardiovascular pleiotropic effects, which are mediated by activation of its nuclear receptor in cardiomyocytes and vascular endothelial cells, as well as regulating the renin–angiotensin–aldosterone system, adiposity, energy expenditure, and pancreatic cell activity [31]. Christakos et al. published a comprehensive review of the pleiotropic effects of vitamin D [32], in which they discussed its metabolism and molecular mechanisms of action, including its pleiotropic effects on cancer, the cardiovascular system, and the immune system.

### 1.6. Hypovitaminosis Increases Vulnerability to Diseases—Causality

Causality can be evaluated using Hill’s criteria in a biological system [33]. The criteria appropriate for vitamin D include the strength of association, consistency, dose–response relationship, biological plausibility, coherence of evidence, experiment, and analogy. As discussed by Doll in 2002, confounding factors and bias must also be considered [34]. He noted that these are not criteria but rather factors that aid in optimizing testing for a nutrient. Observational studies have provided most of the evidence supporting Hill’s criteria. Studies using Hill’s criteria to evaluate causality for vitamin D and various health outcomes will be discussed for some of the health outcomes considered in this work.

Cohort studies have strongly suggested that hypovitaminosis D is associated with the initiation and worsening of diseases [12]. Most studies have confirmed that VDD increases vulnerability to acquiring diseases and developing complications. In addition, once an acute infection is acquired, the vitamin D concentration will decrease rapidly [35]. Unless supplemented, its concentration in the blood will be reduced, prolonging recovery and increasing the risk of developing complications [11,36].

The approach taken in this review is to identify the health outcomes associated with the greatest risk of death in the U.S., and then discuss the evidence supporting the idea that vitamin D could reduce the risks of incidence and death, as well as assess whether the disease outcomes are causally linked to vitamin D status. After that, the new Endocrine Society vitamin D guidelines are discussed. The significance of the analysis in this review is that, based on observational studies rather than clinical trials, raising serum 25(OH)D concentrations above 30 ng/mL would greatly reduce the incidence and mortality rates for 8 of the 10 leading causes of death in the U.S., as well as many other diseases and adverse pregnancy and birth outcomes.

### 1.7. Rationale for the Present Study

Considering the relationship between medical systems and health/disease, organizations rely on RCTs to evaluate the effectiveness of pharmaceutical drugs and the risks of adverse health outcomes. Most vitamin D RCTs conducted to date have been based on guidelines for pharmaceutical drugs; enrolled participants with relatively high 25(OH)D concentrations; given the vitamin D treatment arm relatively low vitamin D doses, as well as giving or permitting the control group to also take vitamin D supplements; and analyzed the results based on intention to treat. As a result, few RCTs have reported beneficial effects associated with vitamin D supplementation.

Observational studies regarding vitamin D have been largely based on serum 25(OH)D concentrations. They also generally include participants with various 25(OH)D concentrations. They have a few drawbacks, such as diminished findings for vitamin D effects when the mean follow-up period becomes too long due to regression dilution associated with changing concentrations [10]; however, this problem can be overcome by comparing outcomes with mean follow-up periods with respect to the measurement of variables such as 25(OH)D concentration [37,38]. The goal of this review is to develop the case for basing vitamin D recommendations on the best available evidence from a variety of approaches, including RCTs, observational studies, Mendelian randomization (MR) studies, mechanistic studies, and evaluations of causality using Hill’s criteria for causality in a biological system [33]. This review will also guide and encourage researchers to design and conduct better vitamin D RCTs.

## 2. Health Benefits of Vitamin D

The health outcomes discussed in this review are presented for 8 of the 10 leading causes of death in the U.S. for 2021 and 2022 [39]. They are, in descending order, heart disease, cancer, COVID-19, stroke, chronic lower respiratory diseases, Alzheimer’s disease (AD), diabetes mellitus, and kidney disease.

### 2.1. Cardiovascular Disease

According to the American Heart Association, CVD accounted for 928,741 deaths in the U.S. in 2020 [40]. The percentages of deaths due to type of CVD were coronary heart disease, 41.2%; stroke, 17.3%; other CVD, 16.8%; hypertension, 12.9%; heart failure, 9.2%; and arterial diseases, 2.6%. In 2022, 702,880 people died from heart disease [41]. The global burden of CVD for 2021 has been estimated at 67 million (95% CI, 61–73 million) incident cases and 19 million (95% confidence interval [CI], 18–21 million) deaths [42].

Vitamin D is associated with cardiovascular benefits, including potential protective effects against heart disease, as it influences calcium homeostasis and gene transcription, supporting myocardial contractility and reducing the risks of cardiac hypertrophy and atherosclerosis [43,44]. Systematic reviews and meta-analyses of RCTs have indicated that vitamin D supplementation improves several cardiovascular risk factors, including a significant increase in HDL cholesterol and reductions in triglycerides and systolic blood pressure [45]. Other studies have suggested supplementation may help heart failure patients [46].

Hypertension is an important risk factor for CVD, especially if associated with other CVD risk factors [47]. Another study using data from the U.K. Biobank evaluated the associations between serum 25(OH)D concentrations, vitamin D supplementation, and CVD mortality among adults with hypertension [48]. In fully adjusted models, serum 25(OH)D concentrations between 25 and 50 nmol/L compared to >75 nmol/L were associated with HR = 1.71 (95% CI, 1.22–2.40) for all-cause mortality rate and HR = 1.87 (95% CI, 1.55–2.27) for CVD mortality. Serum 25(OH)D concentrations < 50 nmol/L compared to >75 nmol/L were associated with HR = 1.97 (95% CI, 1.15–3.39) for all-cause mortality rate and HR = 1.42 (95% CI, 0.70–2.91) for CVD mortality. In a fully adjusted model, vitamin D supplementation was associated with HR = 0.76 (95% CI, 0.61–0.94) for all-cause mortality and HR = 0.75 (95% CI, 0.54–1.03) for CVD mortality.

An open-label vitamin D supplementation study conducted in Canada demonstrated that raising serum 25(OH)D concentrations above 40 ng/mL could lower blood pressure (BP) [49]. The study involved 8155 participants with a mean age of 56 ± 15 years and mean BMI of 27 ± 6 kg/m^2^. Of the total, 592 participants were hypertensive at baseline. For the entire group, vitamin D supplementation at baseline was 1600 ± 2500 IU/day, rising to 5200 ± 4300 IU/day, and the mean serum 25(OH)D concentration rose from 35 ± 15 ng/mL to 45 ± 16 ng/mL. At the end of follow-up (12 ± 3 months later), 71% of hypertensives at baseline were no longer hypertensive.

In an analysis of 40 hypertensive cases not taking BP medications and 80 hypertensives taking BP medications, decreases in mean systolic BP were not significantly different (−18 ± 19 mmHg and −14 ± 21 mmHg, respectively; *p* = 0.25), with similar results for diastolic BP. For 187 pre-hypertensive participants (diastolic BP 130–139 mmHg) not taking BP medications, the changes in systolic BP were −3 ± 16 mmHg; meanwhile, for 374 pre-hypertensives taking BP medications, the mean change in systolic BP was −1 to 1.3 mmHg. Thus, this study demonstrated a significant effect of vitamin D supplementation raising serum 25(OH)D concentrations above 40 ng/mL, leading to lower blood pressure in middle-aged hypertensives, but not others. This is an important clinical treatment effect. However, the results may not apply to other individuals, such as those who are older or with higher BMI.

According to a meta-analysis reported in 2019, RCTs have not shown that vitamin D supplementation reduces the risk of CVD [50]. However, the D-Health RCT conducted in Australia from 2014 to 2020 did find reductions in CVD events [51]. The vitamin D treatment arm participants were given 60,000 IU of vitamin D_3_ per month. For the entire set of participants, the reduction in major cardiovascular events (MACEs) with vitamin D supplementation was not significant (HR = 0.91; 95% CI, 0.81–1.01); however, it was significant for participants taking CV drugs (HR = 0.84; 95% CI, 0.74–0.97).

Low levels of HDL cholesterol (HDL-C) (<40 mg/dL) are strongly associated with increased risks of coronary and peripheral arterial diseases [52]. A meta-analysis of 57 observational studies and two cohort studies found that high vs. low 25(OH)D concentrations were associated with an 18% reduction in HDL-C (OR = 0.82; 95% CI, 0.76–0.89) [53].

A retrospective, observational, nested case–control study evaluated the effects of vitamin D supplementation on the risk of myocardial infarction and all-cause mortality for patients with VDD who received care at the Veterans Health Administration from 1999 to 2018 [54]. Cases and controls were matched using a propensity score-weighted Cox proportional hazard model. In the comparison of 10,014 treated subjects who achieved 25(OH)D concentrations > 30 ng/mL, compared to 2942 untreated subjects with 25(OH)D concentrations < 20 ng/mL, the HR for all-cause mortality rate was 0.61 (95% CI, 0.56–0.67; *p* < 0.001) and the HR for myocardial infarction was 0.73 (95% CI, 0.55–0.96; *p* = 0.02).

**Figure 1:**
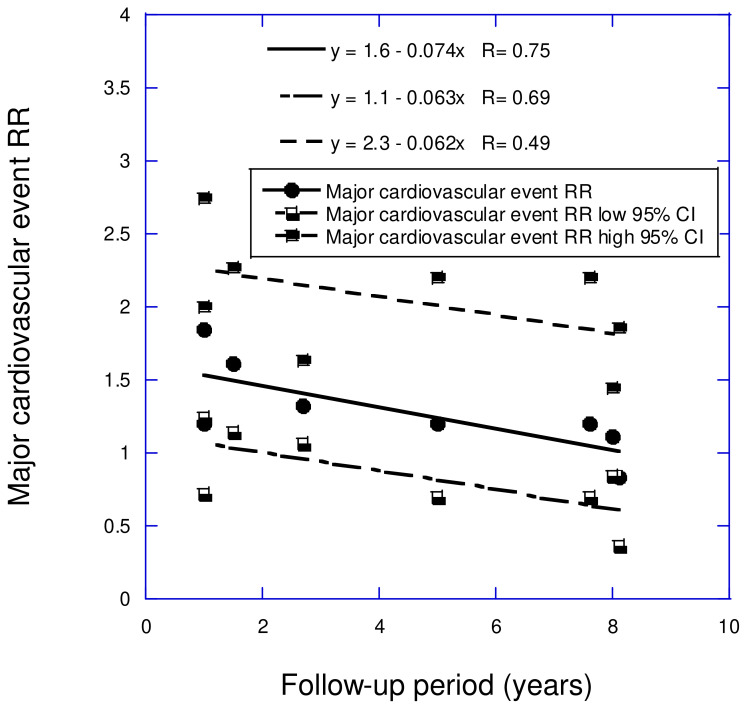
The relative risk of major cardiovascular events (MACEs) for low vs. high serum 25(OH)D concentrations (mostly >30 vs. <20 ng/mL) versus the mean follow-up period [37]. The two shortest follow-up period papers were those of de Metrio [55] and Beska [56], which are considered closest to the actual effect of 25(OH)D concentration against MACE. This figure is from an open-access article distributed under the terms and conditions of the Creative Commons Attribution (CC BY) license (https://creativecommons.org/licenses/by/4.0/, accessed on 10 December 2024).

A study on the effect of the follow-up period on the relative risk of MACE concerning low vs. high serum 25(OH)D concentrations was recently published [37]. The comparisons of serum 25(OH)D concentrations varied from <9 vs. >9 ng/mL to <30 vs. >30 ng/mL. As shown in Figure 1, the regression fit to the data indicated that the risk increased by 50–60%.

MR studies evaluate causal relationships between risk factors and health outcomes. They involve randomizing participants in large databases according to some of their alleles in the vitamin D metabolic pathway to generate a “genetically instrumented 25(OH)D concentration score” for comparison with health outcomes. With large numbers of participants, it is expected that factors affecting 25(OH)D concentrations, such as vitamin D supplementation and solar UVB exposure, will be averaged out. It has been demonstrated that a nonlinear approach with many such genetic scores is a more sensitive approach. An article reported a nonlinear MR analysis of the effect of VDD on CVD risk using data from the U.K. Biobank [57]. It was estimated that correcting VDD to above 75 nmol/L would reduce the risk of CVD by 6% (95% CI, 2–10%). Using this figure for the U.S., the number of CVD deaths that could have been prevented in 2020 was 56,000 (95% CI, 19,000–93,000).

### 2.2. Stroke

**Figure 2:**
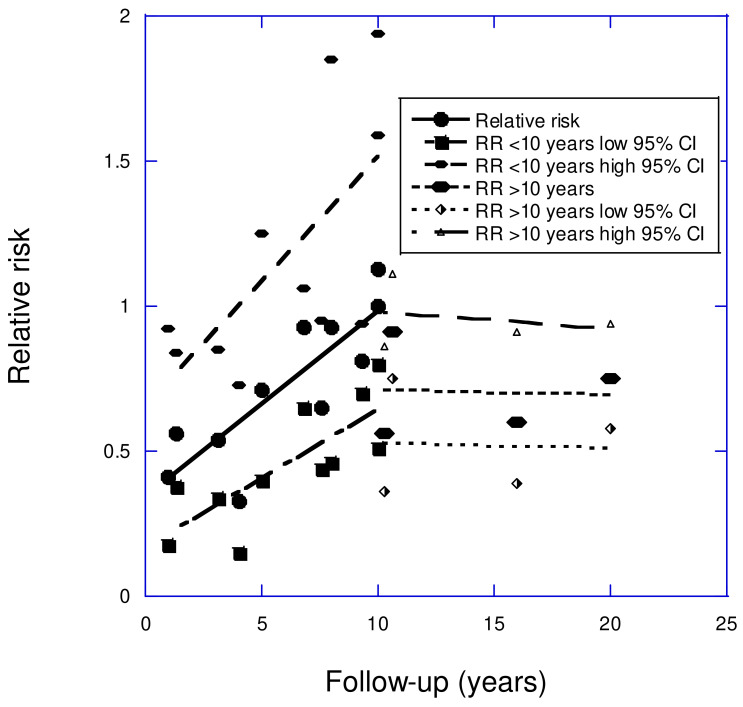
Plot of relative risk of stroke versus years of follow-up concerning high vs. low 25(OH)D concentrations (mostly >30 vs. <20 ng/mL) [37]. The papers with the two shortest follow-up periods are those of Zittermann et al. [61] and Anderson et al. [62], which are considered closest to the effect of 25(OH)D concentration against stroke. This figure is from an open-access article distributed under the terms and conditions of the Creative Commons Attribution (CC BY) license (https://creativecommons.org/licenses/by/4.0/, accessed on 10 December 2024).

Stroke accounted for 160,264 deaths in the U.S. in 2020 [58]. Observational studies have found that the incidence of stroke is inversely correlated with serum 25(OH)D concentration [37]. Many relevant studies have compared the risk of stroke concerning >30 vs. <20 ng/mL. This review analyzed the effect of follow-up time on stroke incidence using studies included in two standard meta-analyses [59,60]. For stroke, a good linear fit to the data for follow-up periods of 1–10 years was observed: RR = 0.34 + (0.065 *×* follow-up [years]), *r* = 0.84, adjusted *r*^2^ = 0.67, *p* < 0.001 (see Figure 2).

It has been argued that the preponderance of evidence supports the claim that vitamin D reduces the risk of stroke outcomes in a causal manner, as evaluated concerning the criteria for causality in a biological system outlined by Hill in 1965 [33]. The only criterion not satisfied is experimental verification through an RCT. However, it must be noted that the beneficial effects of vitamin D against stroke occur at a 25(OH)D concentration of 20 ng/mL, thus requiring the participants in an RCT to initially have concentrations below 20 ng/mL, which is very difficult to achieve in Western developed countries.

### 2.3. Cancer Prevention and Survival

According to the American Cancer Society, the numbers of new cancer cases in 2024 will be 1,029,080 for males and 972,060 for females, while the numbers of cancer deaths will be 322,800 for males and 288,920 for females [63]. The leading types of cancer for males are prostate, lung and bronchus, colorectal, urinary bladder, melanoma of the skin, and kidney and renal pelvis cancers. The first three have the highest mortality rates, followed by pancreas, liver, and intrahepatic bile duct cancers. For females, the top five types of cancer are breast, lung and bronchus, colorectal, uterine corpus, and melanoma of the skin. For deaths, pancreas cancer replaces melanoma in the top five.

Globally, there were an estimated 19.3 million cancer cases and 10.0 million cancer deaths in 2020 [64]. The most common types of cancer, in descending order, were female breast, lung, colorectal, prostate, and stomach cancers. The cancers with the highest numbers of deaths were lung, colorectal, liver, stomach, and female breast cancers.

The evidence that vitamin D can reduce the risk of cancer incidence and mortality rates is robust. A recent review noted that ecological studies have found inverse correlations between solar UVB radiation dose indices and incidence and/or mortality rates for over 20 types of cancer [28]. Solar UVB is a proxy for 25(OH)D concentration. The association between solar UVB dose and cancer incidence was weaker than that with cancer mortality rates. A likely reason for this is that many mechanisms can cause cancer but few reduce cancer mortality. Vitamin D reduces angiogenesis around tumors, which is required to deliver nutrients to the tumors, and it reduces metastasis into the surrounding stromal tissue, which is generally required for mortality.

**Figure 3:**
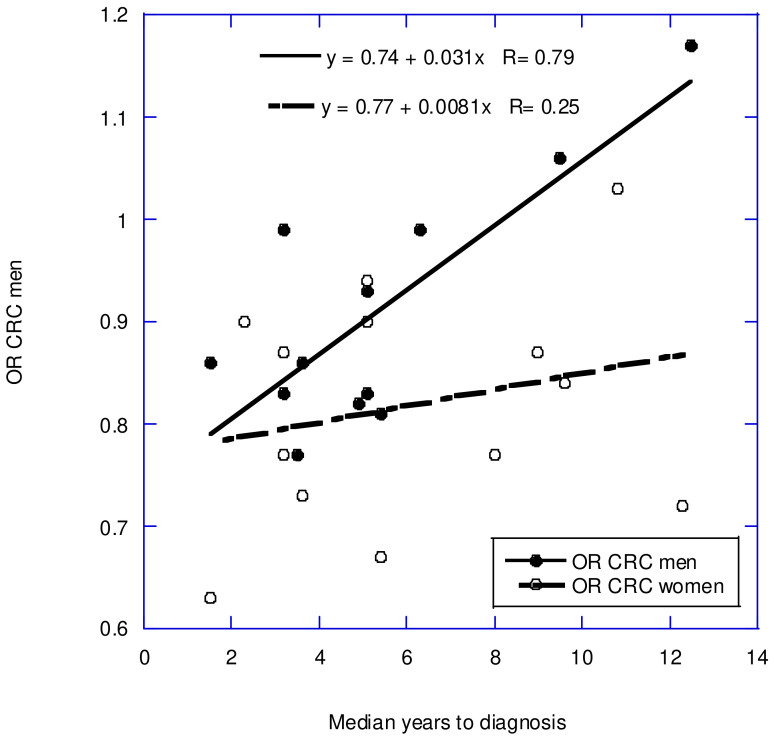
Odds ratio (OR) for colorectal cancer concerning high vs. low 25(OH)D concentrations against median years to diagnosis for data from men and women used in the study of McCullough et al. [27], as shown in [28]. This figure is from an open-access article distributed under the terms and conditions of the Creative Commons Attribution (CC BY) license (https://creativecommons.org/licenses/by/4.0/, accessed on 10 December 2024).

Prospective cohort studies have found inverse correlations between serum 25(OH)D concentrations and the incidences of several types of cancer. However, as published, these studies have not fully demonstrated the beneficial effects of higher concentrations due to changes in serum 25(OH)D concentrations during the follow-up period. A study conducted in Norway found that the correlation coefficient, *r*, for serum 25(OH)D concentrations measured in 2668 participants 14 years apart was 0.42 [65]. A meta-analysis of colorectal cancer incidence concerning serum 25(OH)D concentrations in prospective cohort studies found that, for each 10 ng/mL increase in 25(OH)D concentration, the risk of colorectal cancer was 19% lower in women (RR = 0.81; 95% CI, 0.75–0.87) and 7% lower in men (RR = 0.93; 95% CI, 0.86–1.00) [27]. However, when RR was plotted vs. the mean follow-up period, it was found that the regression fit to the data for men was RR = 0.74, while that for women was RR = 0.77 [28]. Men had a 2.6 times higher rate of change in RR concerning the follow-up period than women (see Figure 3).

The observational study approach has also been used to assess the effects of vitamin D supplementation on breast cancer risk. In an analysis of breast cancer incidence vs. serum 25(OH)D concentration [66], findings were obtained from two vitamin D RCTs [67,68] and the GrassrootsHealth.net volunteer cohort. Multivariate Cox regression analysis revealed that women with 25(OH)D concentrations ≥ 60 ng/mL had an 80% lower risk of breast cancer than those with concentrations < 20 ng/mL (HR = 0.20; 95% CI, 0.05–0.82; *p* = 0.03).

RCTs have also provided limited support for vitamin D supplementation in reducing cancer risk. The largest RCT to study the effects of vitamin D supplementation on the risk of cancer was the VITAL study [15]. This study enrolled over 25,000 participants in 2012–2014, randomly assigning half to take 2000 IU/day of vitamin D3, while the other half was assigned a placebo. The mean baseline all-year 25(OH)D concentrations of those in the vitamin D_3_ treatment arm (for those who provided values) were 29.7 ng/mL for males and 32 ng/mL for females. The mean year-one 25(OH)D concentrations for those in the vitamin D treatment arm were 39.7 ng/mL for males (N = 395) and 43.6 ng/mL for females (N = 441). All participants were permitted to take 600 IU/d (800 IU/d for those over 70 years) of vitamin D, and the participants were followed for a median time of 5.3 years. When analyzed by intention to treat, the HR for cancer incidence was 0.96 (95% CI, 0.88–1.06; *p* = 0.47). However, for those with BMI < 25 kg/m^2^, HR = 0.76 (95% CI, 0.63–0.90) and, for Black people with a mean 25(OH)D concentration of 24.9 ng/mL, HR = 0.77 (95% CI, 0.59–1.01).

In the VITAL trial [15], a significant reduction in the risk of advanced cancers was found for those in the vitamin D supplementation arm compared with the placebo arm (HR = 0.83; 95% CI, 0.69–0.99). Those with BMI < 25 kg/m^2^ had a significantly reduced risk (HR = 0.62; 95% CI, 0.45–0.86), while those with BMI = 25–30 kg/m^2^ or >30 kg/m^2^ did not (HR = 0.89; 95% CI, 0.68–1.17 and HR = 1.05; 95% CI, 0.74–1.49, respectively) [69].

A recent post hoc analysis of a vitamin D RCT conducted in Japan investigated survival for digestive tract cancers [70]. Those in the vitamin D group received 2000 IU/d vitamin D_3_. Eighty of the 392 patients were p53-immunoreactive, and 9 of the 54 patients in the p53-immunoreactive group treated with vitamin D had a relapse or death during 5 years of follow-up, compared to 14 of 26 in the placebo group (HR = 0.27; 95% CI, 0.11–0.61; *p* = 0.002). In the non-p53-immunoreactive group, vitamin D supplementation had no effect. This exciting finding needs further study, including whether it applies to other types of cancer.

There is a large body of literature regarding how vitamin D reduces the risk of cancer [28], and important mechanisms are still being discovered. A recent article described how vitamin D regulates microbiome-dependent cancer immunity [71], resulting in greater immune-dependent resistance to transplantable cancers and augmented responses to checkpoint blockade immunotherapies. This resistance was attributable to the activity of vitamin D on intestinal epithelial cells, which alters the microbiome composition in favor of *Bacteroides fragilis*.

### 2.4. Immune System Support and COVID-19

Vitamin D supports immune function by enhancing innate and adaptive immunity. It boosts antimicrobial peptides such as cathelicidin through 1,25-dihydroxyvitamin D binding to the vitamin D receptor [72]. It has been shown that the Toll-like receptor activates a vitamin D-mediated human antimicrobial response in macrophages, killing *Mycobacterium tuberculosis* [73]. There is also evidence that cathelicidin can deactivate viruses [74], including SARS-CoV-2 [75].

Vitamin D modulates T cells by promoting regulatory T cells while suppressing inflammatory Th1 and Th17 cells [76]. Vitamin D reduces the risk of cytokine storms due to an over-response to viral infection, resulting in greater severity of diseases such as COVID-19 [12]. VDD increases susceptibility to respiratory infections, including SARS-CoV-2 and autoimmune conditions [76,77]. Higher serum 25(OH)D concentrations reduce the risk of viral infectious diseases in general [78] and COVID-19 in particular [79,80], as well as community-acquired pneumonia [81].

Supplementation has shown potential in reducing hospitalization rates and improving outcomes in infected patients [77]. Vitamin D supplementation and adequate vitamin D status also reduce the risks of diseases caused by bacteria and viruses, such as pneumonia [81] and COVID-19 [79,80]. Adequate 25(OH)D concentrations are also linked to reduced incidences of autoimmune diseases and allergic reactions, underscoring their protective effects on the immune system. While the Endocrine Society guidelines recommend supplementation to prevent VDD, they may not account for increased needs during illness or in individuals with chronic inflammatory conditions.

Vitamin D was proposed to reduce the risk of COVID-19 in March 2020 [82]. Evidence presented in support of this suggestion included the fact that higher UVB doses were associated with reduced case fatality rates during the 1918–1919 pandemic influenza in the U.S. [83] and a clinical trial found that vitamin D supplementation reduced the risk of influenza type A in school children [84]. This suggestion turned out to be correct in terms of reduced the risks of SARS-CoV-2 infection [79] and COVID-19 incidence [80], as well as COVID-19 severity and death [85].

SARS-CoV-2 vaccinations were associated with increased excess death rates in many countries [86]. Meanwhile, the use of vitamin D to reduce the risk and severity of COVID-19 was not promoted but instead discouraged due to the development of mRNA “vaccines” to prevent SARS-CoV-2 infection. The U.S. Food and Drug Administration granted emergency use authorization (EUA) for these “vaccines” on 11 December 2020 [87]. Such emergency use authorizations are issued if only no adequate and approved alternatives are available [88]. As a result, the use of vitamin D and several re-purposed drugs to prevent or treat COVID-19 was severely curtailed.

Reducing the risk of COVID-19 can also reduce the risks of other diseases. A recent study based on data from the U.K. Biobank found that having COVID-19 significantly increased the risk of MACE [89]. For those hospitalized for COVID-19, the HR for MACE was 3.85 (95% CI, 3.15–4.24). A possible mechanism explaining this observation is that SARS-CoV-2 infection at the level of the vessel wall potentially destabilizes vulnerable plaques and renders the endothelium more prone to thrombus formation.

Vitamin D likely reduces the risks of many childhood viral diseases. Before the widespread use of vaccinations for childhood viral diseases, such diseases had peak seasonality in late winter and early spring; this was the case for measles [90], mumps [91], rubella [92], respiratory syncytial virus [93], and several others [94]. Winter-spring is the coldest season of the year in mid-latitudes, as well as the season of lowest absolute humidity [95] and 25(OH)D concentrations [3,96]. Cold temperature increases the risk of viral infection due to constriction of the respiratory tract’s capillaries, which restricts the respiratory tract’s epithelial cells from fighting viruses at the first opportunity [97]. Many mechanisms, such as the induction of human cathelicidin, are innate responses controlled by vitamin D [73]. Promoting vitamin D supplementation might also reduce the need for childhood vaccinations, especially for viral infectious diseases that are more common in winter and spring.

### 2.5. Chronic Lower Respiratory Diseases

In 2021, more than 15 million Americans (6.4%) reported that they had been diagnosed with chronic lower respiratory disease, including chronic obstructive pulmonary disease (COPD) [98]. Major risk factors include tobacco smoking, occupational and environmental exposures, respiratory infections, and genetic factors [98]. Based on the Global Initiative for Chronic Obstructive Lung Disease’s fixed ratio, the prevalence of COPD in 2019 was estimated at 392 million (95% CI, 313–488 million) in those aged 30–79 years [99].

There is mounting evidence that higher serum 25(OH)D concentrations are associated with a lower risk of COPD. A recent article reported results on the incidence of COPD concerning serum 25(OH)D concentrations based on data from the U.K. Biobank, with a median follow-up period of 12.3 years [100]. For participants with a baseline 25(OH)D concentration <31.7 nmol/L, compared to concentrations from 51.8 to 64.6 nmol/L, the adjusted HR = 1.23 (95% CI, 1.16–1.31). For COPD-specific death, the adjusted HR = 1.57 (95% CI, 1.03–2.40). An MR study based on European data found an inverse causal association between genetically predicted 25(OH)D concentration and the risk of COPD [101]. Each standard deviation of 25(OH)D concentration increase was associated with a 57% reduced risk of COPD (OR = 0.43; 95% CI, 0.28–0.66).

One of the mechanisms by which vitamin D reduces the risk of COPD may be by reducing inflammation. A hospital-based case–control study in China compared variables for 101 COPD patients and 202 controls [102]. Serum 25(OH)D concentrations were lower in COPD patients (adjusted OR = 0.86; 95% CI, 0.74–0.99; *p* = 0.04). All inflammation-related variables were higher in COPD patients than in controls, including CRP, TNF-α, MCP-1, IL-6, and IL-1β. The values for the variables increased with COPD grade according to forced expiratory volume in 1 s.

### 2.6. Alzheimer’s Disease and Dementia

The number of people in the U.S. with clinical AD in 2020 was estimated at 6.1 million (95% CI, 5.9–6.4 million) people [103]. The U.S. census-adjusted prevalences of clinical AD were 10% among non-Hispanic whites, 14% among Hispanics, and 18.6% among non-Hispanic African Americans [103]. An article estimated the number of people worldwide across the AD continuum as 32 million with AD, 69 million with prodromal AD, and 315 million with pre-clinical AD [104]—this represents 22% of all persons aged 50 and above.

Vitamin D plays a significant role in brain health, cognition, and mood regulation, with emerging evidence supporting its therapeutic potential across various mental and neurological disorders. Adequate 25(OH)D concentrations are associated with improved cognitive function [105,106] and mood stability [107], particularly in vulnerable populations. Vitamin D supplementation has shown promise in enhancing mood and reducing depressive symptoms, with studies indicating improved clinical outcomes in patients receiving vitamin D alongside antidepressants [108].

Additionally, vitamin D deficiency—being prevalent globally—has been associated with cognitive decline in conditions such as schizophrenia [109] as well as AD and dementia [38]. The neuroprotective effects of vitamin D are noted particularly in aging populations, where it may help to mitigate cognitive decline through mechanisms involving neuroinflammation and neurotrophic factors [110]. A comprehensive review of the mechanisms through which vitamin D reduces the risk of AD was published in 2023 [111]. Vitamin D also supports sleep health, improving sleep quality and duration, especially in young adults and those affected by depression [112,113,114].

**Figure 4:**
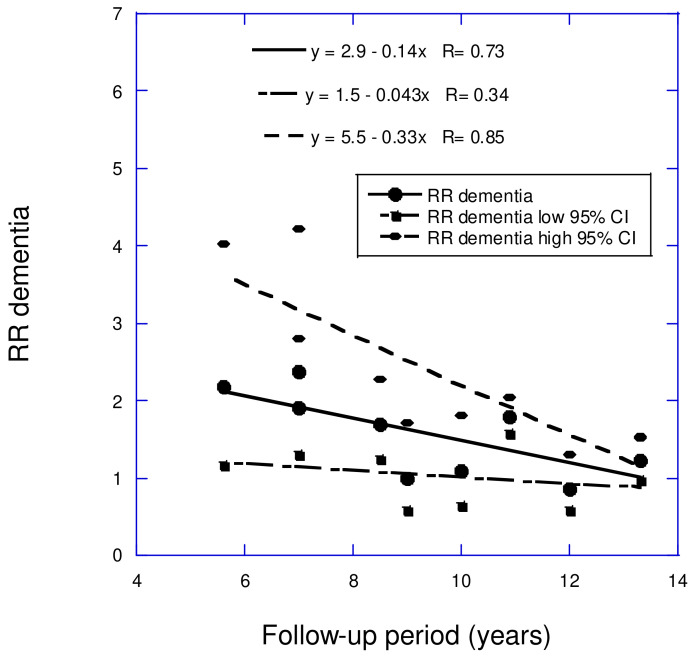
Scatter plot of relative risk (RR) against low vs. high 25(OH)D concentrations (mostly <20 vs. >30 ng/mL) for dementia from [38]. The data for the two shortest follow-up periods were from the studies of Littlejohns et al. [115], Kiderman et al. [116], and Van Lent et al. [117], which are considered the most accurate. This figure is from an open-access article distributed under the terms and conditions of the Creative Commons Attribution (CC BY) license (https://creativecommons.org/licenses/by/4.0/, accessed on 10 December 2024).

**Figure 5:**
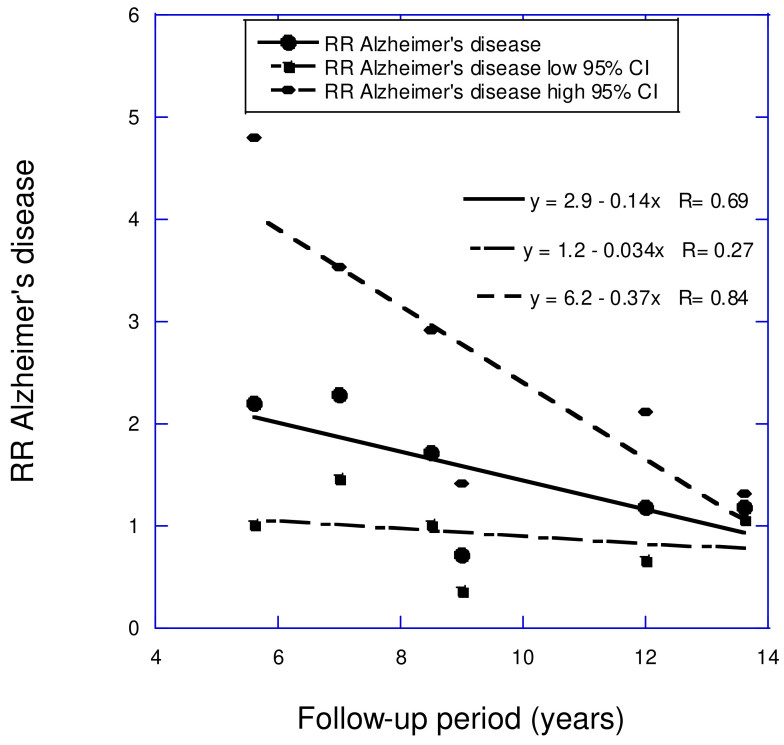
Relative risk (RR) for AD against low vs. high 25(OH)D concentrations (mostly <20 vs. >30 ng/mL) according to the mean follow-up period [38]. The two shortest follow-up period data are from the studies of Littlejohns et al. [115] and Melo van Lent et al. [117], which are considered the most accurate. This figure is from an open-access article distributed under the terms and conditions of the Creative Commons Attribution (CC BY) license (https://creativecommons.org/licenses/by/4.0/, accessed on 10 December 2024).

Prospective cohort studies have evaluated the effect of low vs. high serum 25(OH)D concentrations on the risk of adverse brain health. A recent review examined how the follow-up period affected the results of nine cohort studies focused on all-cause dementia and six studies on AD concerning vitamin D deficiency [38]. The mean follow-up periods were between 3 and 13 years. For all-cause dementia, the comparison was mainly for <20 vs. >20 ng/mL. For AD, the comparison of 25(OH)D concentrations for the shortest three follow-up periods was for <10 vs. >20 ng/mL, while for the longest three follow-up period studies, the comparison of 25(OH)D concentrations was for <20 vs. >20 or >30 ng/mL. For all-cause dementia and AD, for low vs. high serum 25(OH)D concentrations, the linear regression fits were RR = 2.9 − 0.14 *×* years (*r* = 0.73, *p* = 0.02) and RR = 2.9 − 0.14 *×* years (*r* = 0.69, *p* = 0.13), respectively (see Figure 4 and Figure 5). The finding that the regression fit to the data for AD was not significant was attributed to having fewer studies in the analysis (6 for AD versus 8 for dementia), as well as AD accounting for about 70% of dementia cases.

In addition, MR studies also support vitamin D’s role in reducing the risk of AD [118] and dementia [119]. As it would be challenging to conduct an RCT to evaluate the effect of vitamin D on the risk of such outcomes, the results of these studies—in combination with prospective studies and an understanding of the mechanisms—are the best evidence for the effect of vitamin D status on the risk of these outcomes [18]. An analysis of the evidence concerning Hill’s criteria for causality in a biological system [27] would support a causal relationship between higher vitamin D status and reduced risk of dementia and AD.

### 2.7. Type 2 Diabetes Mellitus

An analysis of the data from NHANES revealed that, in 2017–2018, the prevalence of diabetes mellitus (DM) in the U.S. was 14%, with about 4% being undiagnosed [120]. This value was up from 10% in 1999–2000. In 2015, it was estimated that there were 415 million (95% CI, 340–536 million) people aged 20–79 years living with DM, and 5 million deaths were attributed to DM globally [121].

Vitamin D plays a multi-faceted role in managing T2DM, influencing metabolic control, insulin resistance, and weight management. Research has indicated that vitamin D deficiency is linked to increased insulin resistance and pancreatic dysfunction, which can exacerbate T2DM [122]. Vitamin D enhances insulin receptor transcription and glucose transport, potentially reducing insulin resistance. A systematic review of RCTs showed significant improvements in insulin resistance in T2DM patients following vitamin D supplementation among subgroups, including those receiving high-dose vitamin D, the non-obese, vitamin D-deficient individuals, and those with well-controlled HbA1c [123].

However, systematic reviews have shown mixed results regarding the effects of vitamin D on metabolic syndrome parameters, with benefits in glycemic control observed primarily in deficient individuals. At the same time, other studies have suggested that the relationship between 25(OH)D concentration and metabolic health may not be causal [124,125].

The Vitamin D and Diabetes (D2d) study was an RCT regarding the effects of vitamin D supplementation on progression from pre-diabetes to T2DM [16]. Participants in the vitamin D treatment arm were given 4000 IU/day of vitamin D_3,_ while those in the control arm were given a placebo. After a median period of 2.5 years, an analysis of the results regarding the intention to treat showed no benefit associated with vitamin D supplementation. However, in a subsequent analysis based on the achieved serum 25(OH)D concentration in the vitamin D treatment arm, such a benefit was observed [126]; in particular, the risk reduction for those who achieved 40–50 ng/mL compared to 20–30 ng/mL was 52%, and for those who achieved >50 ng/mL it was 71%.

An analysis of the data from NHANES evaluated the risk of death concerning serum 25(OH)D concentrations for adults with DM [127]. A total of 6326 adults with DM were identified between 2001 and 2014, and they were followed until 31 December 2015. A total of 55,126 person-years of follow-up revealed 2056 deaths, and the mean follow-up period was 8.7 years. The adjusted HR for all-cause mortality rate for 25(OH)D concentration >75 nmol/L compared to <25 nmol/L was 0.58 (95% CI, 0.43–0.83), while that for 25(OH)D concentration between 25 and 50 nmol/L was 0.70 (95% CI, 0.55–0.89). This suggests that the all-cause mortality rate reduction for >75 nmol/L vs. 25–50 nmol/L is 14%.

A Danish study utilizing blood test results from the Copenhagen General Practitioners laboratory involved 222,311 individuals, of whom 7652 developed T2DM during follow-up periods from one to eight years [128]. Using 20 ng/mL as the reference 25(OH)D concentration, the HR for T2DM increased in a quasi-linear fashion to 2.0 (95% CI, 1.8–2.1) for 25(OH)D = 10 ng/mL and decreased in a nonlinear fashion to 0.55 (95% CI, 0.50–0.60) above 40 ng/mL.

### 2.8. Chronic Kidney Disease

The data from NHANES have been used to estimate the prevalence of chronic kidney disease (CKD) in the U.S. [129]. The prevalence of CKD was 13.3% (13.0–14.4%) in 2015–2018. Age-adjusted prevalence rates by race/ethnicity were non-Hispanic Blacks, 16.3 ± 2.1%; Hispanics, 14.3 ± 1.4%; and non-Hispanic Whites, 12.5 ± 1.4%. The rates by race/ethnicity were consistent with variations in serum 25(OH)D concentration by race/ethnicity for non-supplement users in the period 2001–2010 [130].

Globally, it has been estimated that 844 million individuals had CKD in 2017 [131], and CKD caused 4.6% (95% CI, 4.3–5.0%) of global deaths in 2017 [132]. More information regarding the burden of CKD can be found in a recent review [133].

An article has reviewed how vitamin D could increase survival in CKD [134]. Figure 1 in that article outlined how activation of the vitamin D receptor could reduce mortality from CKD, with the associated mechanisms including effects on cardiac hypertrophy, atherosclerosis, vascular calcification, thrombosis, immune status, and tumorigenesis, in addition to lowering parathyroid hormone (PTH) concentrations in cardiac, vascular, metabolic, hematology, and immunology contexts.

A recent article reported findings regarding all-cause and cardiovascular mortalities in older people with chronic kidney disease (CKD) [135]. Data were obtained for 3230 CKD patients who were followed-up for a median period of 6.2 years. Compared with those in the deficiency group (<50 nmol/L), those in the insufficient (50–75 nmol/L) and sufficient (≥75 nmol/L) groups were significantly associated with lower all-cause mortality (HR = 0.83; 95% CI, 0.71–0.97 and HR = 0.75; 95% CI, 0.64–0.89, respectively) and cardiovascular mortality (HR = 0.87; 95% CI, 0.68–1.10; and HR = 0.77; 95% CI, 0.59–1.00, respectively).

### 2.9. Bone and Oral Health

Vitamin D is crucial for calcium absorption and bone mineralization, and its role in reducing rickets is well known [136]. A systematic review found that vitamin D supplementation increased bone mineral density at femoral neck, lumbar spine, and total hip sites [137]. A meta-analysis of seven RCTs found that supplementation with 800 IU/day of vitamin D_3_ plus 1000 mg/day of calcium significantly reduced the risk of hip fracture (OR = 0.69; 95% CI, 0.58–0.82) [138].

Controlled clinical trials conducted in the 1950s showed that vitamin D supplementation reduced the incidence of dental caries in children by about 50% [139]. Vitamin D status is inversely associated with periodontal disease inflammation [140].

### 2.10. Autoimmune Diseases

Vitamin D has gained attention for its potential in managing autoimmune diseases, mainly through high-dose protocols such as the Coimbra Protocol, which modulates immune responses to improve outcomes [141]. This protocol involves administering high doses of vitamin D_3_—often exceeding 35,000 IU daily—under strict supervision, with studies showing it to be safe (regarding calcium metabolism and renal function). By regulating immunity through the inhibition of Th1 and Th17 responses while enhancing Treg activity, vitamin D helps to reduce inflammation and maintain immune balance [142].

The action mentioned above is particularly beneficial in preventing over-active immune reactions, which are commonly observed in autoimmune diseases and allergies [76]. It has shown promise in improving conditions such as systemic lupus erythematosus [143]. Its immunomodulatory effects make vitamin D a valuable tool in managing inflammation and supporting overall immune health. The VITAL RCT found that supplementation with 2000 IU/day of vitamin D_3_ significantly reduced the incidence of autoimmune diseases [144]. Despite promising evidence, some studies have suggested that autoimmune diseases might be a consequence—and not a cause—of vitamin D deficiency [145]. The Endocrine Society’s guidelines may underestimate necessary doses for individuals with vitamin D resistance [1], underscoring the need for personalized protocols.

### 2.11. Pregnancy, Birth, and Infancy Outcomes

An estimated 13.4 million (95% CI, 12.3–15.2 million) babies were born preterm (<37 weeks) globally in 2020 (9.9% of all births; 95% CI, 9.1–11.2%) [146]. Rates of gestational diabetes in the U.S. in 2019 were 63.5 (95% CI, 63.1–64.0) per thousand live births [147]. Pre-eclampsia rates vary globally from 2% to 8% [148]. Eclampsia, a severe form of pre-eclampsia, was associated with 0.3% of live births in the U.S. from 2009 to 2017 [149].

Vitamin D status is crucial during pregnancy, influencing fetal skeletal development and reducing risks such as gestational diabetes, pre-eclampsia, and preterm birth [150,151]. A key study demonstrating the benefits of vitamin D during pregnancy was performed in Iran [22], which comprised a stratified randomized field trial investigating the effectiveness of a prenatal vitamin D deficiency screening and treatment program. This study included 900 pregnant women from two health centers. While 800 women at one center were given vitamin D supplementation, the women at the second center were not supplemented and served as controls.

Women at one center with 25(OH)D concentrations between 10 and 20 ng/mL were randomly selected to receive one of four vitamin D_3_ supplementation schedules, varying from 50,000 IU/week for six weeks to a single intramuscular dose of 300,000 IU vitamin D_3_ and a monthly dose of 50,000 IU/month until delivery. Meanwhile, women with 25(OH)D concentrations below 10 ng/mL were randomly selected to receive one of four vitamin D_3_ supplementation schedules: 50,000 IU/week for 12 weeks; 50,000 IU of oral D_3_ weekly for a total duration of 12 weeks plus a monthly maintenance dose of 50,000 IU of D3 until delivery; an intramuscular dose of 300,000 IU vitamin D_3_ every six weeks; two 50,000 IU doses/week for six weeks; or intramuscular administration of 300,000 IU of D_3_ every 6 weeks for two doses plus a monthly maintenance dose of 50,000 IU of D_3_ until delivery.

In the comparison between the two centers, those with baseline 25(OH)D concentrations between 10 and 20 ng/mL did not present significant differences in terms of risk of gestational diabetes or preterm delivery. However, those in the treated center showed significant differences in pre-eclampsia (OR = 0.5; 95% CI, 0.3–0.9). Meanwhile, for those with baseline 25(OH)D concentrations below 10 ng/mL, significant reductions were found at the treated center in terms of pre-eclampsia (OR = 0.3; 95% CI, 0.2–0.5), gestational diabetes (OR = 0.5; 95% CI, 0.3–0.9), and preterm delivery (OR = 0.3; 95% CI, 0.2–0.5). Thus, this study demonstrated that severe-to-moderate vitamin D deficiency is causally associated with increased risks of adverse pregnancy and birth outcomes. It would be impossible to conduct a similar RCT in Western developed countries, as it is considered unethical not to give participants in the control arm a minimal amount of vitamin D (generally 400 to 800 IU/day).

However, an open-label vitamin D supplementation trial was conducted in pregnant women to evaluate the effect of serum 25(OH)D concentration on the risk of preterm birth [152]. Over 1000 pregnant women visiting an urban medical center in South Carolina, USA, were enrolled in the study. Their serum 25(OH)D concentrations were measured, and they were given free vitamin D supplements and counseled on achieving >40 ng/mL. Preterm birth rates were significantly lower for those who achieved >40 ng/mL than those with concentrations <20 ng/mL (OR = 0.41; 95% CI, 0.21–0.72). Reductions were also significant for those who achieved 30–20 ng/mL (OR = 0.53; 95% CI, 0.31–0.91). The results were largely independent of race or ethnicity.

Adequate concentrations in newborns prevent nutritional rickets and other developmental issues. Extensive research has highlighted the role of vitamin D in pregnancy, emphasizing its importance for maternal and fetal health [153]. Despite these benefits, the current Endocrine Society guidelines focus primarily on bone health, potentially overlooking the critical roles of vitamin D in prenatal care [1,22].

### 2.12. All-Cause Mortality

The all-cause mortality rate concerning serum 25(OH)D concentration was analyzed using individual participant data from 26,916 European consortium members with a mean follow-up period of 10.5 years [154]. The adjusted HRs (with 95% CI) for mortality in the 25(OH)D groups with 16–20, 12–16, and <12 ng/mL were 1.15 (95% CI, 1.00–1.29), 1.33 (95% CI, 1.16–1.51), and 1.67 (95% CI, 1.44–1.89), respectively.

### 2.13. Vitamin D Deficiency-Associated Deaths and Their Prevention

An analysis of deaths by day of the year from 1979 to 2004 in the U.S. revealed that rates were 30% higher near the end of the year than near the end of summer [4]. Evidence supporting the hypothesis that a significant fraction of the increased deaths in winter could have been reduced though higher 25(OH)D concentrations was reviewed [5]. Diseases with pronounced winter increases in mortality rates in the U.S. include respiratory tract infections and CVD. At the same time, smaller effects were found in digestive system diseases, as well as endocrine and metabolic diseases.

Table 1 presents the findings regarding the age rates of adverse health effects for several leading causes of death in the U.S., including mortality rates for all causes, CVD, and COVID-19; incidence rates for cancer; and prevalence of DM. As can be seen from the table, rates increase with age, with the highest rates occurring above the age of 65. However, rates begin to rise above the age of 45 years. These data imply that vitamin D status contributes to the risk of adverse health effects even in middle age (if not sooner). Thus, we disagree with the 2024 Endocrine Society guidelines, which recommend that persons between 18 and 75 years should not have their serum 25(OH)D concentrations measured [1], as many of these individuals would benefit from knowing their 25(OH)D concentrations such that they could take measures to achieve the desired concentration [155], especially those who are poor vitamin D responders. It has been shown that serum 25(OH)D concentrations can vary by ±20% under the same vitamin D intake due to genetic variations in the vitamin D metabolic pathway [156].

### 2.14. Racial Disparities

A recent article presented data on American individuals’ mean serum 25(OH)D concentrations from 2001 to 2018 [160]. The data were obtained during eight NHANES surveys. During the 2017–2018 survey, the mean 25(OH)D concentrations by race were as follows: Mexican American, 57.3 nmol/L (95% CI, 54.5–60.1); non-Hispanic White, 81.0 nmol/L (95% CI, 77.6–84.4); non-Hispanic Black, 54.7 nmol/L (95% CI, 51.7–57.8); and other, 66.6 nmol/L (95% CI, 63.7–69.5). In that period, the percentages of VDD [characterized as 25(OH)D below 50 nmol/L (20 ng/mL)] were: Mexican American, 40.2% (95% CI, 34.5–46.0%); non-Hispanic White, 12.2% (95% CI, 8.7–15.7%); non-Hispanic Black, 53.1% (95% CI, 46.7–59.5%); and other, 26.9% (95% CI, 23.2–30.6%). Thus, it would be expected that lower 25(OH)D concentrations among Mexican Americans and non-Hispanic Blacks would translate to higher rates of adverse health outcomes, as has been previously observed [161]. The adverse health effects that were found to be significantly higher for Blacks compared to Whites, which may be attributed to disparities in serum 25(OH)D concentrations, included several types of cancer, COVID-19, all-cause mortality, and adverse pregnancy outcomes.

### 2.15. Higher Vitamin D Doses and Serum 25(OH)D Concentrations from Recommendations

There have been several recommendations regarding vitamin D doses and serum 25(OH)D concentrations. Table 2 lists the vitamin D recommendations issued by government agencies, organizations, and experts from 1997 to 2024. It was not until 2013 that sufficient evidence that vitamin D supplementation should aim to achieve a level of 30–50 ng/mL was available from observational studies [162,163]. RCTs do not supply much evidence for guiding recommendations due to their poor designs, enrolling participants with above-average 25(OH)D concentrations, providing the vitamin D treatment arm with low vitamin D doses, and analyzing the results according to the intention to treat [19,20]. Thus, observational studies provide the best evidence for recommendations.

Experts recommend higher vitamin D doses and serum 25(OH)D concentrations than government agencies and conventional health organizations. This difference is because government agencies and conventional health organizations are largely controlled by those with pharmaceutical and medical treatment interests who profit from treating disease rather than preventing disease. Conventional nutrition adheres to population-based guidelines, such as Dietary Reference Intakes, primarily aiming to prevent deficiencies and maintain baseline health. By contrast, orthomolecular nutrition employs individualized, often high-dose nutrient therapies to achieve therapeutic effects and optimize health outcomes [170]. In addition, mainstream medicine interprets the dictum of the Hippocratic Oath—“First do no harm”—to mean that it may be better to do nothing than to intervene and cause more harm than good. However, it is apparent from the studies discussed in this review that many lives may have been lost due to not raising serum 25(OH)D concentrations through vitamin D supplementation.

Few risks are associated with high-dose vitamin D supplementation and high serum 25(OH)D concentrations. The greatest concern is the development of hypercalcemia, which has an adverse effect. The symptoms of hypercalcemia are well known and, once it is diagnosed, it can be resolved by stopping vitamin D supplementation and waiting.

In response to the new Endocrine Society guidelines 2024 [1], Holick published a counter-manuscript with suggestions [169], highlighting its discordance with 20 findings regarding the relative reduction in clinical outcomes for serum 25(OH)D concentrations. One was for >60 ng/mL (pre-eclampsia), two were for >50 ng/mL (pre-diabetes to T2DM and breast cancer incidence), six were for >40 ng/mL (autoimmune disorders, Cesarean section births, dental caries in infants, relapse and death due to digestive cancers, multiple sclerosis, and premature births) and seven were for >30 ng/mL (cancer mortality, cardiovascular mortality, colon cancer, COVID-19 mortality, and respiratory distress syndrome, osteomalacia, and upper respiratory tract infection) [169]. The wide range of health outcomes showing improvements above 30 ng/mL indicates that 30 ng/mL should be the absolute minimum recommended serum 25(OH)D concentration. The number of outcomes improved above 40 ng/mL further justifies recommending 40 ng/mL as the minimum 25(OH)D concentration, covering a broader group of disorders.

If one considers Holick’s suggestions to be better, then it can be concluded that the vitamin D dose required to achieve concentrations above 40 ng/mL in most people needs to be determined. A review in 2020 presented a table of vitamin D doses and serum 25(OH)D concentrations in selected clinical trials [171]. Some of the articles with higher vitamin D doses and the 25(OH)D concentrations achieved are included in Table 3. As can be seen, 4000 IU/day of vitamin D_3_ supplementation is required to increase the serum 25(OH)D concentration to about 50 ng/mL, even for mildly obese participants. Thus, measuring the achieved 25(OH)D concentration is often useful. Table 3 illustrates the serum 25(OH)D concentrations achieved following different doses of vitamin D. As can be seen from the table, the changes in serum 25(OH)D concentration tend to present large standard deviations, which are due to differences in BMI, baseline serum 25(OH)D concentration, and genetic variations along the vitamin D metabolic pathway (which can be in the range of ±20%) [156].

### 2.16. Different Serum 25(OH)D Concentrations Are Needed to Overcome Diverse Disorders

A serum 25(OH)D concentration above 20 ng/mL is adequate to support the needs of the musculoskeletal system, such as skeletal physiology and neuromuscular coordination, thus preventing falls and fractures [14]. However, other systems require higher serum concentrations of 25(OH)D for their biological functions [178]; for example, to reduce the risks of CVDs and metabolic disorders such as diabetes, insulin resistance, autoimmune diseases, and certain cancers [162].

**Figure 6:**
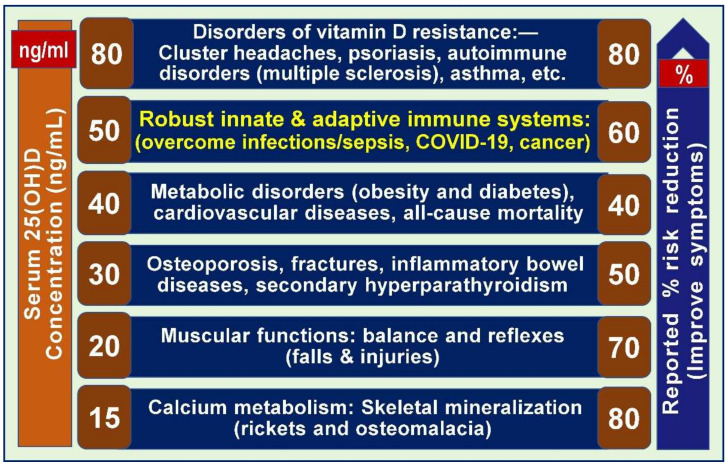
Calculated serum 25(OH)D concentrations needed to overcome different groups of conditions and disorders and the reported average (percentage) improvements/responses in primary clinical outcomes. The cumulated data from many outcome-based vitamin D-related clinical trials (both observational and RCTs) studies are summarized (Wimalwansa et al., 2024) [12,18]. This figure is from an open-access article distributed under the terms and conditions of the Creative Commons Attribution (CC BY) license (https://creativecommons.org/licenses/by/4.0/, accessed on 10 December 2024).

The optimal serum 25(OH)D concentrations for achieving beneficial health outcomes thus vary, depending on the specific disease entity and affected tissue. Emerging data have confirmed the importance of maintaining varied serum 25(OH)D concentrations to effectively counteract and reduce the risks of different diseases, as illustrated in Figure 6, while minimizing complications linked to hypovitaminosis D [9,178,179]. For disorders beyond those affecting the musculoskeletal system, serum 25(OH)D concentrations should be kept above 40 ng/mL [178]. Examples of such conditions include cancer [180,181], T2DM [126], and all-cause mortality [154,182,183].

Maintaining serum 25(OH)D concentrations above 40 ng/mL can significantly reduce the risks associated with various diseases [178]. Evidence suggests that doubling serum 25(OH)D concentrations in the population—for example, from 20 ng/mL to 40 ng/mL—could lead not only to decreased risks of diseases [11] but also to a notable reduction in all-cause mortality, including premature deaths [184,185]. To benefit a larger number of people, it is recommended to maintain serum 25(OH)D concentrations between 40 and 70 ng/mL—this will overcome most of these disorders [11,23]. Figure 6 illustrates the varying steady-state serum 25(OH)D concentrations required to prevent or mitigate the effects of common diseases.

**Figure 7:**
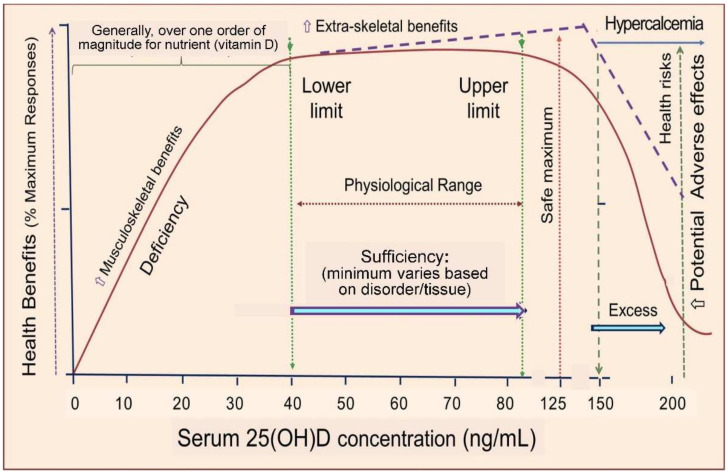
The dose–response for vitamin D with the associated health benefits. When the vitamin D [25(OH)D] level reaches sufficiency for a given tissue/system, no further benefits will be obtained (i.e., providing more would not provide additional physiological benefits). The broken red line illustrates that the beneficial effects of vitamin D could continue without causing hypercalcemia when high doses are administered under close medical supervision. Unlike pharmaceutical agents, nutrient response curves are narrower by about half of an order of magnitude (Wimalwansa et al., 2024) [18]. This figure is from an open-access article distributed under the terms and conditions of the Creative Commons Attribution (CC BY) license (https://creativecommons.org/licenses/by/4.0/, accessed on 10 December 2024). The broken purple line in the upper right corner of Figure 7 represents those with rare indications (i.e., resistance to standard therapy), such as drug-resistant migraine and cluster headaches, psoriasis, asthma, etc. These groups of patients require significantly higher serum 25(OH)D concentrations to be maintained to achieve vital benefits, for example, between 80 and 150 ng/mL [12]. As with the Coimbra protocol, benefits can be obtained without demonstrable adverse effects when appropriately treated under medical supervision. Hypercalcemia generally does not manifest under 150 ng/mL [12].

These data substantiate the necessity of higher thresholds for specific disease categories, particularly among older individuals and those with a high body mass index [99]. Neglecting such clinical practice and clinical trials can lead to poor health outcomes. Figure 7 illustrates the dose–response curve of vitamin D.

In addition, the doses, frequency of administration, and duration of many vitamin D RCTs were inappropriate. As a result, their endpoints and conclusions are considered unreliable. A lack of appreciation for the phenomenon illustrated in Figure 7 will lead to failures in expected clinical outcomes. This is illustrated in several recent large-scale vitamin D RCTs—some designed to fail. As with piggyback studies in pharmaceutical trials, their primary endpoints focused on pharmaceutical agents, not vitamin D [11]. In studies where vitamin D was not the primary interventional agent or was given at an insufficient dose, thus failing to raise the serum 25(OH)D level above the expected threshold in the circulation, as illustrated in Figure 6, outcome failure is expected. Therefore, the data and conclusions from such trials cannot be relied upon for drug approvals, clinical guidelines, or policy decision making [18].

### 2.17. High-Dose Vitamin D and Vitamin D Resistance

High-dose vitamin D therapy has gained attention for its potential in addressing vitamin D resistance (VDRES), where standard doses are ineffective. Research has suggested that VDRES can result from genetic mutations affecting vitamin D receptor (VDR) signaling and environmental factors such as infections [186,187,188]. Acquired vitamin D resistance syndromes are becoming more common. In addition, lifestyle factors such as diet and other micronutrient imbalances can contribute to the need for high-dose vitamin D supplementation to effectively overcome resistance and maintain adequate 25(OH)D concentrations [186].

Genetic polymorphisms in the vitamin D system can lead to low responsiveness and autoimmune diseases, while infections and toxins may inhibit VDR signaling, requiring higher doses for therapeutic effects [187,188]. Clinical applications of high-dose protocols, such as 1000 IU/kg daily, have been effective in treating autoimmune conditions such as multiple sclerosis, and high doses (e.g., 50,000 IU) have led to improvements in insulin sensitivity in populations with metabolic syndrome [189]. When properly monitored, these high-dose protocols can be safe and help patients with underlying health conditions [141].

The Endocrine Society’s guidelines caution against high doses due to toxicity concerns; however, this approach is overly conservative, particularly when considering autoimmune diseases that have limited effective treatments. Further, it is stated that, “Based on the panel’s best estimates of treatment effects in adults aged 50 years and older, the panel judged that any desirable effects of intermittent, high-dose vitamin D (compared to lower-dose, daily vitamin D) are likely trivial, while the anticipated undesirable effects are likely to be small.” The significant group of patients exposed to several drugs daily are asking this group of experts, “why are you going to become trivial when suggesting the above recommendation for us taking so many medicines daily?” Under such challenging conditions, where traditional therapies often fail to achieve sustained results, the potential benefits of higher doses may outweigh the risks. Patients with autoimmune diseases often have few options, making it critical to explore more aggressive interventions, such as higher vitamin D dosing strategies, which may offer more effective relief and long-term improvement.

## 3. Recommendations for Prevention of Vitamin D Deficiency

Serum 25(OH)D concentrations can be increased in several ways: solar UVB exposure, vitamin D supplementation, food fortification with vitamin D, and diet, including animal products [190]. Vitamin D production from solar UVB exposure is more efficient when the solar elevation angle exceeds 45° [191]; however, vitamin D will still be produced at lower angles, albeit at lower rates, and exposing more skin helps to increase its production.

Vitamin D supplementation is the most efficient way to increase 25(OH)D concentrations, which can be achieved throughout the year and in a controlled manner. The case has been made that 2000 IU/day (50 µg/day) is the minimum appropriate dose for many people with normal weight, permitting them to achieve around 30–40 ng/mL with minimal safety concerns [9]. This dose can be taken daily, weekly (15,000 IU), or monthly (60,000 IU). Compliance might be better with weekly or monthly doses. Low-dose supplementation can take several months to achieve steady-state concentrations in those with vitamin D deficiency [192]. Thus, taking large (bolus) doses for the first week or two is recommended to shorten the time required to reach a steady state [23].

Food fortification with vitamin D has been suggested for increasing serum 25(OH)D concentrations [193]. RCTs have been performed on vitamin D fortification of bread, orange juice, mushrooms, cheese, yogurt, fluid milk, powdered milk, eggs, edible oils, and breakfast cereal [194]. Finland increased vitamin D food fortification from 2003 to 2011 [195]. In 2003, it was recommended that vitamin D be added to fat spreads at a concentration of 10 µg/100 g and fluid milk products at 0.5 µg/100 g. These values were doubled in 2010. As a result, the mean serum 25(OH)D concentrations among non-supplement users increased by 20 nmol/L (95% CI, 19–21 nmol/L) between 2000 and 2011 for daily fluid milk consumers and by about 15 nmol/L for fat spread consumption. The mean serum 25(OH)D concentration increased from 48 nmol/L to 65 nmol/L, which could also be related to increased vitamin D supplementation. A subsequent analysis based on a Northern Finland Birth Cohort 1966 study revealed that the mean serum 25(OH)D concentration increased from 54.3 nmol/L in 1997 to 64.9 nmol/L in 2012–2013 [196]. These increases were attributed to vitamin D supplements and consumption of fluid milk but not fat spread. Consequently, vitamin D deficiency rates were cut in half.

In the U.S., milk is fortified with vitamin D. It would be worthwhile to consider fortifying foods preferred by African Americans, who tend to be lactose intolerant and consume less milk, as well as Hispanics, who typically have lower 25(OH)D concentrations than White people. The most efficient way to increase 25(OH)D concentrations is through vitamin D supplementation. This can be achieved in a measured way, allowing the desired 25(OH)D concentrations to be achieved, provided that serum 25(OH)D concentrations are measured due to individual variations in vitamin D dose–25(OH)D concentration relationships (see, e.g., [156]).

## 4. Critiques of the Endocrine Society’s Vitamin D Guidelines

The Endocrine Society (2024) [1] recommends against screening serum 25(OH)D concentrations in adults aged 18–74 years and fails to provide any diagnostic threshold for the determination of vitamin D status. The “empiric vitamin D”, according to the “technical remarks”, “include daily intake of fortified foods, vitamin formulations that contain vitamin D, and/or daily intake of vitamin D supplement (pill or drops)”. Previous Endocrine Society (TED) guidelines in 2011 [2], Central European guidelines published in 2023 [197], and many other related documents published by various medical societies worldwide also suggested 25(OH)D concentration measurements for the prevention or treatment of vitamin D deficiency (VDD), aiming for a level of 30–40 ng/mL (75–100 nmol/L). Meanwhile, some have suggested an optimal level of 40–60 ng/mL [11].

Michael Holick already published his response to the new TES [169]. He pointed out that these guidelines focus on RCTs and ignore all other clinical trials reporting associations [11]. Table 1, in his response, presented the percentage reduction in 20 clinical outcomes concerning suggested serum 25(OH)D concentrations, based largely on observational studies. The reductions were reported from 25% to 100% for high vs. low concentrations, mostly above 30–40 ng/mL vs. <20 ng/mL. There was one outcome for which the threshold was >60 ng/mL, two for >50 ng/mL, six for >40 ng/mL, and eight for >30 ng/mL. Prospective observational studies generally provide the best clinical evidence regarding the beneficial effects of vitamin D on health outcomes due to the limitations of RCTs in demonstrating the benefits of vitamin D supplementation [19,20]. Thus, 4000 IU/day is recommended to raise serum 25(OH)D to the 40–70 ng/mL range to achieve added protection against many adverse health outcomes.

The Endocrine Society’s (2024) [1] guidelines on vitamin D have notable limitations. Firstly, the guidelines emphasize bone health and overlook broader benefits such as immune support, cancer prevention, and cardiovascular health. The recommended dosages are conservative, even for maintaining bone health. The recommended 600 IU dosage for children aged 1 year and older and adults up to age 75 is often inadequate in raising circulating concentrations of 25(OH)D above 30 ng/mL. This concentration is necessary to observe health benefits such as reducing the risk of upper respiratory tract infections and type 1 DM in children [198], improving birth outcomes, and lowering the risk of progression from pre-diabetes to T2DM [126]. Despite significant variations in vitamin D metabolism between individuals, personalized supplementation based on genetic and lifestyle factors is also underemphasized.

It has been noted that everyone has a “vitamin D response” based on variations in the alleles of genes involved in the vitamin D pathway. According to a recent article, individuals may possess serum 25(OH)D concentrations up to 20% higher or lower than the average, based on their genetics [156]. This is in addition to other factors that affect serum 25(OH)D concentrations, such as reduced production of vitamin D from solar UVB irradiance [199], seasonal variations [3,96], BMI [200], people of color [161], medications [201], and diet [190]. Thus, the measurement of serum 25(OH)D concentrations can be significant.

Furthermore, the guidelines caution against high doses (above 4000 IU/day) without fully exploring their therapeutic potential, particularly for autoimmune diseases or chronic illnesses, where higher doses are considered safe and effective under medical supervision [166]. The lack of guidance on safely managing high-dose vitamin D therapy limits the practical utility of the guidelines.

The new guidelines also ignore the health benefits of vitamin D supplementation for people between 18 and 75 years old. As shown in Table 1, people in that age range in the U.S. die from diseases for which vitamin D offers some protection. Routine vitamin D testing is not strongly recommended outside specific risk groups, potentially leading to widespread under-diagnosis and missed opportunities for early intervention.

Environmental and lifestyle factors are also addressed insufficiently, such as latitude, pollution, diet, nutrition, and sun exposure, which dramatically influence vitamin D status. Populations in northern latitudes or those who spend most of their time indoors may require more aggressive supplementation yet, the guidelines remain general and conservative.

Many countries that are not among Western developed countries have high rates of VDD. This can occur in the Middle East due to consuming diets based more on vegetables than animal products, wearing concealing clothing, and staying indoors in the hot summer [202]. It has been suggested that in countries with large fractions of the population with VDD, a combination of vitamin D fortification of food and promotion of vitamin D supplementation be recommended to increase serum 25(OH)D concentrations above 30 ng/mL.

In summary, while focusing on ensuring minimal bone health standards, the new guidelines fail to fully leverage vitamin D’s broader health benefits. Considering vitamin D’s wide safety profile, affordability, and therapeutic potential, a more individualized and proactive approach would better serve public health. The new guidelines are based on vitamin D RCTs, which mostly fail to confirm the health benefits of vitamin D supplementation. They ignore hundreds of other clinical research studies that provide convincing evidence of the extra-skeletal benefits of vitamin D associated with proper vitamin D intake. Vitamin D RCTs have been based on guidelines for pharmaceutical drugs and thus do not apply to micronutrients [12,19,20].

As discussed earlier in this review, guidelines that only focus on bone health are inappropriate for nutrients such as vitamin D and are misleading. In the field of micronutrients, observational studies have become an essential type of study mechanism.

## 5. Limitations

Individual observational studies may not apply to other populations: studies conducted in Western developed countries may not apply to developing countries; studies involving men may not be relevant for women; studies of various age groups may not apply to other age groups; those with higher BMI may not achieve the same benefits from vitamin D as those with normal BMI; and studies regarding prevention may not apply to progression and mortality. For example, the mechanisms by which vitamin D helps to prevent cancers differ from those for treatment, as after cancer initiation, angiogenesis around tumors and metastasis become important [28]. Factors limiting vitamin D’s effects, such as co-nutrients (e.g., calcium and magnesium), diet in general, and exercise, are not discussed. A major limitation is that, as RCTs have been conducted based on vitamin D doses rather than serum 25(OH)D concentrations, support from clinical trials is generally lacking in supporting findings from observational studies.

## 6. Clinical Practice Implications

Physicians should familiarize themselves with vitamin D’s roles in preventing and treating major diseases, and patients should be informed about the benefits of vitamin D. Serum 25(OH)D concentrations should be determined for patients most likely to be deficient, or patients can be advised that taking 1000–4000 IU/day of vitamin D_3_ could be beneficial. Patients diagnosed with the diseases discussed in this review should be informed of the existing evidence regarding the role of vitamin D in their disease and advised to take vitamin D_3_ accordingly. All pregnant women should take 2000–4000 IU/day vitamin D_3_ during pregnancy and when breastfeeding.

## 7. Conclusions

Vitamin D is a critical component of the human body, with far-reaching effects on health. It is necessary essential for the entire lifespan, from prenatal to end-of-life stages. Evidence for the beneficial effects of vitamin D comes from observational studies, clinical studies, MR analyses, and studies of mechanisms. RCTs have generally not supported vitamin D’s health benefits due to being based on guidelines for pharmaceutical drugs, enrolling participants with relatively high baseline serum 25(OH)D concentrations, using relatively low vitamin D doses, giving small vitamin D doses to participants in the control arm and permitting them to take additional vitamin D supplements, and analyzing the results based on intention to treat rather than the achieved 25(OH)D concentrations.

Observational studies—primarily prospective cohort studies—provide the best epidemiological evidence for most health outcomes and formed the basis for most of the recommendations in this review. A limitation of prospective cohort studies is regression dilution due to the changing 25(OH)D concentrations of the participants. This limitation can be mitigated by examining the effect of the follow-up period in meta-analyses, as was performed in this review. MR analyses also provide some relevant evidence. Mechanistic studies are helpful in understanding how vitamin D affects various health outcomes, supporting the findings of epidemiological studies.

The main recommendation from this review is that serum 25(OH)D concentrations should be raised above 30 ng/mL, with a suggested range of 40–70 ng/mL. Raising mean 25(OH)D concentrations at the population level would be expected to significantly reduce the incidence and mortality rates for 8 of the 10 leading causes of death in the U.S., as well as adverse pregnancy and birth outcomes.

As healthcare systems prefer evidence from RCTs rather than observational studies, we recommend that additional RCTs be conducted to evaluate the findings from observational studies. To do so, all participants enrolled should have baseline 25(OH)D concentrations below 20 ng/mL; they should be given enough vitamin D_3_ to raise 25(OH)D concentrations above 40 ng/mL, achieving 25(OH)D concentrations after a few months and adjusting the vitamin D doses as required; participants in the control arm should not be given any vitamin D supplements as part of the RCT; and 25(OH)D concentrations should be measured every 6–12 months in order to capture seasonal variations as well as concentrations shortly before any adverse health outcomes.

In addition, regarding the risk of disease, it would help if participants in RCTs were chosen who had reasonably high risks of developing the adverse health outcomes of interest, such as pre-diabetics at risk of T2DM. Furthermore, it is thought that supplementation with co-supplements, such as calcium, magnesium, and vitamin K_2_, may further promote the effects of vitamin D. In that case, all participants should be given the same doses of co-supplements. In RCTs aimed at treating existing diseases, all participants should receive the same standard of care.

## Figures and Tables

**Table 1:** Age dependence of adverse health effects for several leading causes of death in the U.S.

Age Range	All-Cause Mortality Rate (Deaths/ 100,000) in 2022 [39]	CVD * Mortality Rate (Deaths/ 100,000) in 2022 [157]	Cancer Incidence (%) 2017–2019 [63]	COVID-19 Mortality Rate (Deaths/ 100,000) in 2022 [158]	DM Prevalence (%) August 2021– August 2023 [159]
25–34	163		0–49 years 3.5	5	20–39 years, 3.6
35–44	255	65		12	
45–54	453		50–64 years F, 10.8; M, 11.8	30	40–59 years 17.7
55–64	992	251		71	
65–74	1979	541	F, 24.3; M 31.9	158	60+ years 27.3
75–84	4706	495		414	
85+	14,390	698	F, 39.6; M, 41.6	1224	

*, 76% heart disease, 17% stroke; CVD, cardiovascular disease; DM, diabetes mellitus; F, female; M, male.

**Table 2:** Recommendations or suggestions for vitamin D supplementation and serum 25(OH)D concentration for adults.

Year	Organization, Country	Vitamin D Dose (IU/day)	Serum 25(OH)D (ng/mL)	Health Basis	Comments	Reference
1997	Institute of Medicine, USA	200–600 Depending on age		Bones		[164]
2010	Institute of Medicine, USA	600 to 70 years, 800 for >70 years	20	Bones	Based on RCTs	[14]
2011	Endocrine Society, USA	1500–2000	30	Bones, VDD	Insufficient evidence for non-skeletal effects	[2]
2013	International Conference, Experts	800–2000; 1600–4000 for obese	30–50	Non-skeletal effects [162]		[163]
2014	Experts	4000–6000	40–52	Physiological		[165]
2019	Experts	5000–50,000	30–120	Treatment (e.g., psoriasis)		[166]
2023	Experts	Bolus	30–50	Sepsis		[167]
2024	Experts	2000	30–50	VDD		[8]
2024	Experts	2000	30	VDD		[9]
2024	Endocrine Society, USA	600–800 1–18, 75+ years		VDD	Lack of RCTs, Observational studies ignored	[1]
2024	Experts	7000–10,000	40–60	Obese, multi-morbidity		[168]
2024	Experts	1500–2000	30, 40–60 preferred	Skeletal, extra-skeletal effects	Observational studies	[169]
2024	Experts		40–80	Extra-skeletal disease prevention, treatment	Observational studies	[18]

**Table 3:** Selected findings regarding serum 25(OH)D concentrations achieved with higher vitamin D supplementation doses.

Population	Intervention Vitamin D Supplementation (IU/d)	Comparison	Outcome Units (ng/mL)	Reference
62 obese individuals (BMI, 37 ± 5 kg/m^2^); aged 45 ± 12 years; mean baseline 25(OH)D, 20–26 ng/mL	1000, 5000, or 10,000 for 21 weeks in winter	Dose (IU/day), baseline (ng/mL) 1000 IU, 20 ± 6 5000 IU, 27 ± 7 10,000 IU, 23 ± 15	Increments of 25(OH)D 1000 IU, 12 ± 10 5000 IU, 28 ± 10 10,000, 48 ± 20	[172]
39 healthy male athletes; 20 years; BMI, 24; U.K.	5000 for 14 weeks in winter	Placebo	25(OH)D increased from 22 (17–28) to 50 (39–60) vs. 23 (16–28) to 13 (11–20)	[173]
3882 community-based participants, Canada	BMI 22 ± 2 kg/m^2^ Supplementation (IU/day) Base, 2200; Int, 6100 BMI 27 ± 1 kg/m^2^ Base, 2100; Int, 6800 BMI 34 ± 4 kg/m^2^ Base, 1900; Int, 7700 For 6–18 months		BMI 22 ± 2 kg/m^2^ Base, 37 (SD 12); Int, 52 (SD 16) BMI 27 ± 1 kg/m^2^ Base, 35 (SD 11); Int, 50 (SD 15) BMI 34 ± 4 kg/m^2^ Base, 32 (SD 10); Int, 47 (SD 15)	[174]
Long-term hospitalized patients, USA	N = 36, 5000/day for 12 months N = 78, 10,000 IU/day for 12 months		5000 IU, Base 24; Ach, 68 (range 41–95) 10,000 IU, Base 25; Ach, 96 (range 53–148)	[166]
2423 overweight/obese (Mean BMI, 32 [SD 4]) pre-diabetic patients, USA	4000/day, 24 months		Base, 28 (SD 10) Ach, 54 (SD 15)	[175]
30 healthy adults, BMI < 30 kg/m^2^	600, 4000, or 10,000 IU/d of vitamin D_3_ for 6 months		162, 320, and 1289 genes up- or downregulated in white blood cells, respectively	[30]
67 T2DM patients with peripheral neuropathy; BMI, 30 (SD 2) kg/m^2^; Russia	40,000/week, 24 weeks	5000/week, 24 weeks	40,000 IU Base, 16 (SD 8), Ach, 72 (SD 17); 5000 IU Base, 19 (SD 8), Ach, 27 (SD 7)	[176]
2423 overweight/obese prediabetes patients, USA	4000 for 3 years	Placebo	Achieved 25OHD Adverse events, RR = 0.94 (95% CI, 0.90–0.98)	[177]

Ach, achieved; BMI, body mass index; Int, intervention; IU, international unit; RR, relative risk; SD, standard deviation; T2DM, type 2 diabetes mellitus.
